# Evaluating the Effectiveness of Gravity-Assisted Ankle Stress AP Imaging in Detecting Syndesmosis Injuries: A Retrospective Clinical Study

**DOI:** 10.3390/diagnostics15212803

**Published:** 2025-11-05

**Authors:** Bahattin Kemah, Elif Reyyan Çadırcıbaşı, Muhsin Yıldız, Mehmet Salih Söylemez

**Affiliations:** 1 Department of Orthopaedics and Traumatology, Umraniye Training and Research Hospital, 34766 Istanbul, Türkiye; bahattinkemah.md@gmail.com (B.K.); drelifreyyan@gmail.com (E.R.Ç.); dr.muhsinyildiz@gmail.com (M.Y.); 2 Department of Orthopaedics and Traumatology, Medistate Hospital, 34805 Istanbul, Türkiye

**Keywords:** syndesmosis injury, ankle fracture, lateral malleolus fracture, gravity-assisted ankle stress radiography, diagnosis, reliability

## Abstract

**Background:** While gravity-assisted ankle stress AP (GAASA) images have proven effective in evaluating deep deltoid ligament injuries, their efficacy in assessing syndesmosis injuries remains unclear. We aimed to investigate the diagnostic performance of GAASA images in detecting syndesmosis injuries. **Methods:** This study reviewed records of patients aged 16+ with unilateral ankle fractures in a single-center ER from 2022 to 2023. Three orthopedic surgeons evaluated standard AP and lateral X-rays, ankle mortise, and GAASA and bilateral ankle CT images in blinded sessions for syndesmosis injuries. Evaluations were repeated to assess the inter- and intra-rater reliability. **Results:** A total of 121 patients with suspected syndesmosis injuries were included in this study. The average age of the patients was 49.9 ± 16.6 years. Syndesmosis injuries were present in 32.2% of cases. The inter-observer reliability was the highest for GAASA images (κ = 0.701) and mortise radiographs (κ = 0.735), and lowest for CT images (κ = 0.426). GAASA images had a sensitivity of 82% and specificity of 68%. Mortise images had 55% sensitivity and 81% specificity. GAASA images showed better discriminatory power for syndesmosis injuries compared to mortise and CT images. **Conclusions:** GAASA images demonstrated superior sensitivity and better negative predictive values in detecting syndesmosis injuries compared to mortise radiographs and CT images. While GAASA may serve as a useful adjunct for evaluating syndesmosis injuries, its interpretation requires careful clinical correlation, and it should not be considered a replacement for standard imaging in all cases. GAASA may be of particular value in emergency or resource-limited settings where CT is not readily available, offering a practical option for ruling out injury in many patients.

## 1. Introduction

Syndesmosis injuries in ankle fractures not only necessitate definitive surgical intervention but also demand precise surgical management, as they represent inherently unstable injuries. However, due to the complex anatomical structure of the ankle and the intricate nature of associated injuries, no single diagnostic tool—whether dynamic, static, or clinical—has been established as a reliable method for accurately detecting these injuries [1]. Standard anterior–posterior (AP) and lateral radiographs often fail to detect syndesmotic disruption due to the overlap at the distal tibiofibular joint, where separation might not be clearly visualized in certain cases [2]. Bilateral computed tomography (CT) scans can reveal volume changes in the distal tibiofibular joint by comparing the injured side with the intact contralateral side, thereby offering higher sensitivity and specificity than conventional radiographs [3]. Furthermore, CT images obtained under axial or rotational stress have been reported to improve the detection of subtle syndesmotic injuries, providing even greater diagnostic accuracy in selected cases. Despite these advantages, CT scans are often avoided due to concerns about radiation exposure. Moreover, accurate diagnosis using CT requires meticulous volume measurements and a thorough understanding of the technique [4]. Dynamic diagnostic methods typically need to be performed under anesthesia, as they are painful procedures that provoke the separation of the distal tibiofibular joint. When conducted without anesthesia, these tests demand significant patient cooperation. Gravity-assisted ankle stress AP (GAASA) imaging is a highly specific technique used to assess the integrity of both the syndesmosis and the deltoid ligament without the need for anesthesia or significant adjustments to the patient’s orientation [5]. This image can be obtained by applying a gravitational force or undergoing manual external rotation. Since manual external rotation can cause substantial pain, gravitational force is generally better tolerated by patients. To obtain a GAASA radiograph, the patient is positioned in a lateral decubitus position, with the ankle hanging freely and unprotected, 10–20 cm beyond the edge of the X-ray table, allowing gravity to act as the stress force [6,7,8].

The GAASA image, which captures the ankle under the influence of gravitational force, allows the weight of the foot to be transferred from the talus to the distal fibula. The primary anatomical structures preventing lateral displacement of the ankle mortise are deep deltoid ligament fibers, the distal fibula, and the syndesmosis ligament complex connecting the fibula to the tibia [9]. Theoretically, a defect in any of these structures would manifest as separation on the affected side. These radiographs have demonstrated effectiveness in evaluating injuries to the deep deltoid ligament fibers [7]. However, despite reports of their efficacy in assessing syndesmosis injuries, there is a lack of conclusive studies comparing their sensitivity, specificity, positive predictive value, and negative predictive value with CT imaging. The current study aims to investigate the diagnostic performance of the GAASA image in detecting syndesmosis injuries. We hypothesized that the GAASA image would demonstrate superior sensitivity and specificity in detecting syndesmosis injuries compared to traditional mortise X-rays and CT imaging, as gravitational force propagates displacement in the syndesmosis.

## 2. Materials and Methods

After obtaining approval from the Ethics Committee of the Ümraniye Training and Research Hospital (ID: B.10.1.TKH.4.34.H.GP.0.01/259), we retrospectively reviewed the medical records of patients who were referred to the ER for ankle injuries at a single center between 2022 and 2023. This study was conducted in accordance with the principles for human experimentation defined in the Declaration of Helsinki. Written informed consent was obtained from all individuals prior to the application of diagnostic and treatment methods.

The study included patients aged 16 years or older with a unilateral ankle fracture and the necessary medical records needed, including appropriate X-rays, bilateral ankle CT images, and at least 6 months of follow-up data. Patients who underwent either surgical intervention or conservative treatment were included in the study group, as in most cases of ankle or malleolar fractures; the presence of a syndesmosis injury was the critical determinant guiding the decision between operative and nonoperative management. In surgically treated patients, syndesmosis injuries are more often apparent, requiring less additional imaging, whereas in conservatively managed patients, less severe injuries may create diagnostic uncertainty. Therefore, the inclusion of patients who underwent conservative treatment was essential to ensure a comprehensive evaluation of the entire patient population. Patients were excluded from the study if they lacked adequate medical records, had pathological or open fractures, any additional cruris injuries, congenital lower extremity pathologies, new or previous injuries in the same extremity, a history of prior ankle surgery, or had not completed at least 6 months of follow-up.

The following data were collected for each patient: age, side of injury (left or right), gender, and associated injuries. Fractures were classified according to the Weber [10] and Lauge-Hansen [11] classification systems, as well as their anatomical location.

Evaluations were conducted using documents from the hospital’s picture archiving and communication system (PACS). Classifications were carried out by the second and third authors, with any conflicts resolved during a consensus meeting. All images were formatted for a PowerPoint presentation to blind the cases for the three evaluators (raters).

A total of 178 patients with ankle fractures underwent both X-ray and CT scans. However, 57 patients were excluded from the study because their bilateral ankle CT, standard AP and lateral radiographs, mortise radiographs, or gravity-assisted ankle stress AP images were not obtained in accordance with the established protocol. Consequently, the X-rays and CT images of 121 patients were evaluated for the purposes of this study. GAASA images were obtained with the patient in the lateral position with the affected limb facing down. A block was placed proximally to the fibula fracture [12]. The foot was allowed to relax or dangle in an externally rotated position, producing gravitational stress through the ankle fracture. Mortise and standard X-rays were obtained using traditional methods [13].

**Figure 1:**
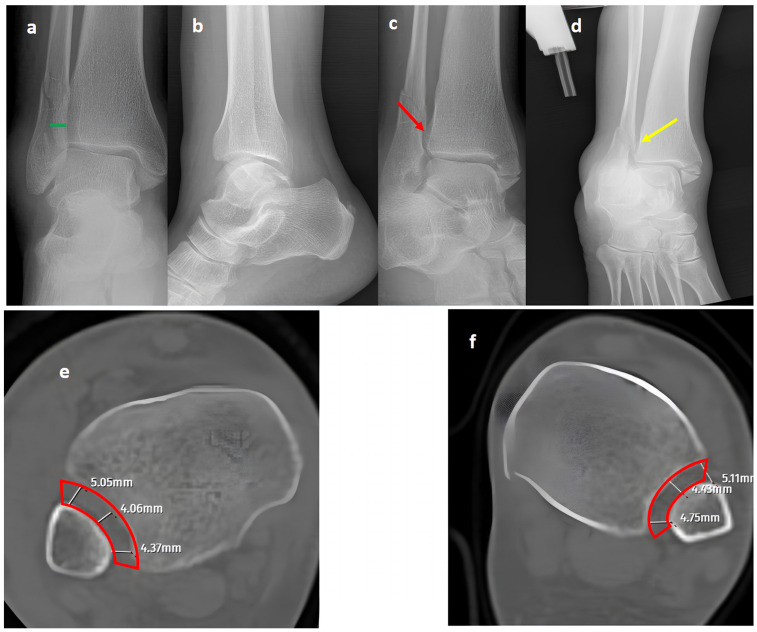
A trimalleolar right ankle fracture. (**a**,**b**) Standard AP and lateral views showing a bimalleolar fracture with a normal distal tibiofibular overlap (green line). (**c**) A mortise view showing an open distal tibiofibular distance (red arrow) indicating syndesmosis injury. (**d**) A GAASA view showing lateral displacement of the talus with a shift in the medial malleolus and distal fragment of the fibular segment. However, the proximal segment of the fibula is in place, indicating that the proximal syndesmosis is intact (yellow arrow). Axial CT images of the injured right ankle (**e**) and the uninjured left ankle (**f**) show measurement of the tibiofibular space (red block bow), which appears similar and within normal ranges, indicating an intact syndesmosis.

**Figure 2:**
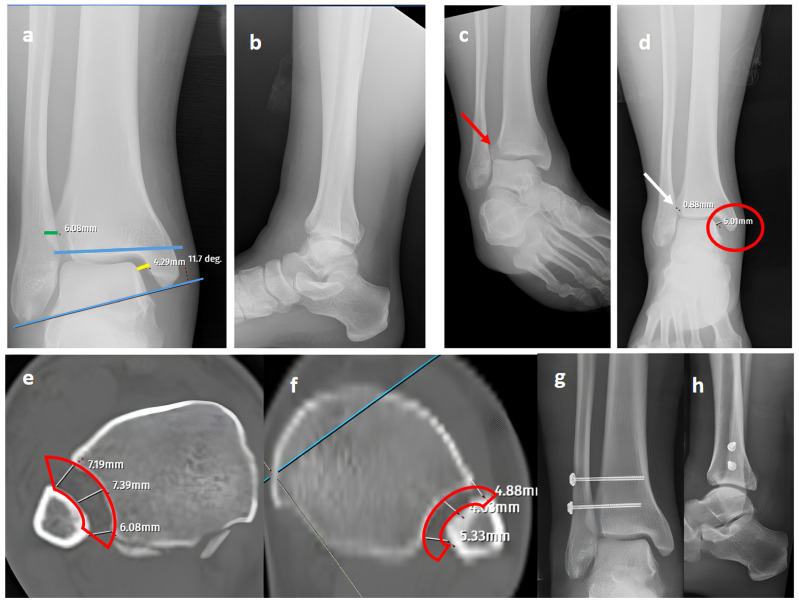
A Maisonneuve (pronation-external rotation IV) fracture. (**a**,**b**) Standard AP and lateral views showing a posterior malleolar fracture with a normal distal tibiofibular (green line) overlap, medial clear space (yellow line), and talocrural angle (blue lines). (**c**) A mortise view showing an obviously widened distal tibiofibular distance (red arrow), indicating syndesmosis injury. (**d**) A GAASA view showing lateral displacement of the talus with widening of the medial clear space (red circle) within physiological limits, indicating that the deltoid ligament is intact. However, widening is still evident for the distal tibiofibular distance due to syndesmosis injury (white arrow). (**e**) A comparison of axial CT images of the injured right ankle and axial CT images of the uninjured left ankle (**f**) reveals obvious distortion of the whole syndesmosis (red block bows) for the right side. (**g**,**h**) Postoperative X-rays showing reduction and fixation of the mortise without the need for the intervention of posterior structures, indicating that the deltoid ligament is intact, consistent with the GAASA image.

**Figure 3:**
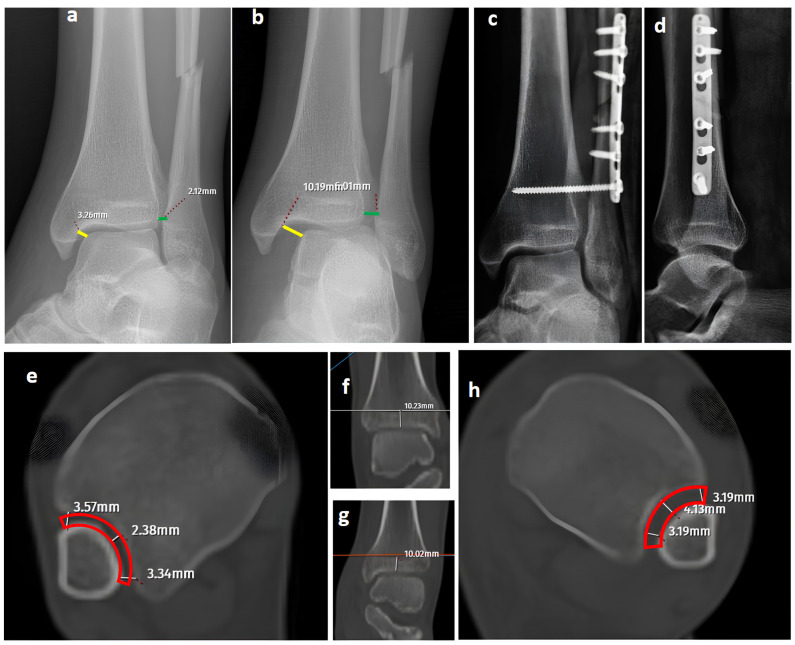
A bimalleolar equivalent (pronation external rotation IV) left ankle fracture. (**a**) A mortise view showing a high lateral malleolar fracture with normal medial clear space (yellow line) and distal tibiofibular space (green line). (**b**) A GAASA image showing widened medial clear space (yellow line) and widened distal tibiofibular distance (green line), indicating syndesmosis injury and deltoid ligament injury concomitantly. (**c**,**d**) Initial postoperative AP and lateral X-rays showing anatomical reduction after fixation of the lateral malleolus and syndesmosis without repair of the deltoid ligament. (**e**–**h**) Axial CT images of both ankles. Although the left-side syndesmosis seems to be widened compared to the right foot, measurements are within normal ranges and do not indicate a syndesmosis injury (red block bows).

Three different sets of images were prepared and evaluated by three orthopedic surgeons with varying levels of experience during three separate sessions (a senior resident surgeon, a staff orthopedic surgeon, and a senior orthopedic surgeon). The image sets included standard AP/lateral radiographs and mortise radiographs, standard AP/lateral radiographs and GAASA images, and standard AP/lateral radiographs and CT axial (10 mm over the plafond) and coronal images. One week prior to evaluating the image sets, all evaluators were informed in a meeting about the radiological findings indicating syndesmosis injuries, including evaluations of the interosseous clear space, tibiofibular clear space, medial clear space, talo-crural angle, and tibiofibular overlap [1] (Figure 1, Figure 2 and Figure 3).

Two senior trauma surgeons and the evaluators jointly reviewed all radiographs, CT images, intraoperative notes, and follow-up X-rays for the patients. This comprehensive assessment was accepted as the definitive diagnosis of all cases of syndesmosis injuries.

In the initial evaluation session, the researchers presented the evaluators with a mixture of three different image sets: standard radiographs and mortise radiographs, standard radiographs and GAASA images, and standard radiographs combined with CT axial and coronal images showing 1 cm proximal to the mortise [14]. For the first two radiograph sets, the evaluators had two options to choose from: syndesmosis injury present or syndesmosis injury not present. Additionally, the raters could select a third option indicating that they would like to request additional CT imaging to make a definitive diagnosis, paired with one of the initial two options. By contrast, for the standard radiograph + CT image set, the evaluators were only provided with two options: syndesmosis injury present or syndesmosis injury not present. The evaluators were blinded to patients’ clinical information. Examiners assessed each image set and determined the presence or absence of syndesmosis injury, providing their assessment in a standardized data collection form. However, no measurements were allowed during the evaluation sessions, and only qualitative interpretation of images was performed. This was performed to mimic real-world clinical practice, where surgeons often must rely on their ability to visually recognize pathological widening or distortion without the benefit of exact measurements. This approach allowed us to assess the examiners’ ability to subjectively identify syndesmosis injuries under practical conditions.

Two weeks later, a second evaluation session was conducted with the same three image sets presented in a different order to evaluate the intra-observer reliability. To determine the overall sensitivity, specificity, positive predictive value, and negative predictive value, the diagnosis selected by a two-thirds majority of the evaluators during the first session for each patient in the radiograph-based assessments was considered the valid diagnosis. This diagnosis was then compared to the definitive diagnosis established through the consensus review process.

Data from the initial session assessed inter-rater reliability across the image sets, while data from the subsequent session evaluated intra-rater reliability. Additionally, the comparison of first-session data to the definitive diagnosis determined the percentage of cases where standard radiographs and mortise radiographs, as well as standard radiographs and GAASA images, were sufficient for diagnosis without further evaluation by experienced surgeons.

### Statistical Analyses

Data were analyzed using SPSS software (Version 26.0, IBM Corporation, Armonk, NY, USA). The inter-observer and intra-observer reliability were assessed for qualitative data using Fleiss’ kappa coefficient and Cohen’s kappa coefficient. A κ value between 0.81 and 1.00 indicated almost perfect agreement; 0.61 to 0.80 indicated substantial agreement; 0.21 to 0.60 indicated moderate agreement, and 0.20 or lower indicates slight agreement. Quantitative variables were expressed as means, medians, and standard deviations, and qualitative variables were expressed as frequencies or proportions. All statistical evaluations were performed using a 95% confidence interval.

*p*-values less than 0.05 were considered statistically significant.

## 3. Results

The average age of the patients included in this study was 49.9 ± 16.6 (16–83) years. While 105 patients had a fracture in the lateral malleolus, 16 (13.2%) did not have a lateral malleolus fracture (Table 1). The most common injury type was an isolated lateral malleolus fracture (*n* = 57, 47.1%), and there was a single case (0.8%) of isolated ligamentous syndesmosis injury without a fracture at any level. The most common fracture level was Weber B, accounting for 63% of cases. According to the Lauge-Hansen classification, the most common type of injury was supination-external rotation type 2 (*n* = 45, 42%) (Table 1).

Syndesmosis injuries were present in 39 patients (32.2%), and 74 patients underwent surgical treatment (61.2%). Syndesmosis injuries were significantly associated with pronation-external rotation injuries, Weber type B lateral malleolus fractures, and isolated lateral malleolus and trimalleolar fractures (*p* = 0.001, 0.033, and 0.01, respectively).

For inter-observer reliability evaluations, substantial agreement was observed for GAASA images (Fleiss κ = 0.701) and mortise radiographs (κ = 0.735) among the evaluators. Surprisingly, the agreement was lowest for CT images (κ = 0.426).

Regarding intra-observer reliability evaluations, the results showed an almost excellent level of agreement for both mortise and GAASA images for the senior resident; by contrast, the staff orthopedic surgeon exhibited a substantial level of agreement (Cohen’s κ = 0.91/0.83 and 0.70/0.73, respectively). However, the senior orthopedic surgeon demonstrated only a moderate level of agreement. Interestingly, intra-observer agreement was limited for CT images across all levels of expertise (Cohen’s κ = 0.15/0.06 and 0.19, respectively) (Table 2).

The need for further imaging, either with mortise or GAASA images, was lowest for the GAASA image set across all evaluators, regardless of their level of experience. Additionally, the need for further radiological evaluation increased with less experience when a CT image was not available (Table 3).

Diagnostic performance differed according to evaluator experience and imaging modality. For senior residents, mortise radiographs yielded the best balance (sensitivity 77%; specificity 81%), while GAASA images provided similar sensitivity (71%) but slightly lower specificity (71%). Staff surgeons achieved their highest sensitivity with GAASA (74%), despite modest specificity (54%), whereas mortise radiographs showed a more balanced profile again (64% sensitivity; 66% specificity). Senior orthopedic surgeons demonstrated improved results with GAASA (77% sensitivity; 67% specificity) compared with mortise radiographs (50% sensitivity; 84% specificity). CT images consistently performed worst across evaluators, with sensitivity below 40% and poor predictive values. When data were pooled, GAASA provided the highest sensitivity (82%) and negative predictive value (89%), whereas mortise radiographs offered the highest specificity (82%). (For more information, see Supplementary Table S1).

The positive likelihood ratio and negative likelihood ratio were 3.7 and 0.2 for GAASA images and 2.8 and 0.5 for Mortise radiographs, respectively, suggesting that GAASA images have better discriminatory power for syndesmosis injury. Conversely, the positive likelihood ratio and negative likelihood ratio were 0.4 and 1.2 for CT image sets, indicating that CT images and standard radiographs had a limited ability to diagnose syndesmosis injury in this study.

## 4. Discussion

Several studies have evaluated various parameters and diagnostic tools to detect potentially unstable syndesmosis injuries with effectiveness. However, the reported results and parameters are highly variable, and many of these techniques are either not widely applicable or require specialized equipment and patient cooperation, or could cause significant discomfort or pain despite their potential diagnostic value [15]. Some of the investigated methods include measuring the surface area of the syndesmosis using CT imaging [16]; weight-bearing X-rays in cases of isolated fibular fractures [17]; 3D SPACE sequences and 2D proton density–weighted MRI for assessing both acute and chronic syndesmosis injuries [18]; high-resolution 3T MRI combined with arthroscopic evaluation and stress testing, where available [19]; conventional ankle CT scans performed with forced external rotation and dorsiflexion [20]; dynamic CT imaging of the syndesmosis under axial loading in different foot positions [21]; and measurement of the tibiofibular distance without applying any external stress [22]. GAASA imaging is an effective tool for evaluating deep deltoid ligament disruption comparable to manual stress radiography in detecting deltoid ligament injuries associated with lateral malleolus fractures, without a significant increase in patient-reported pain scores [23,24]. Moreover, GAASA offers advantages, including lower radiation exposure and the elimination of the need for a physician to apply manual stress during the examination. However, there is limited evidence on its use in the specific evaluation of syndesmosis injuries [1,12,25].

The present study demonstrates that gravity-assisted stress AP (GAASA) imaging is a valuable adjunct to standard radiographic assessment for diagnosing syndesmotic injuries associated with ankle fractures. Our hypothesis—that GAASA imaging would provide superior sensitivity and specificity compared to mortise radiographs and CT imaging—was partially supported. Our findings indicate that GAASA demonstrated superior sensitivity in detecting syndesmosis injuries, while mortise radiographs showed better specificity for excluding these injuries. Therefore, using both specialized radiographic techniques with standard radiographs may optimize diagnostic accuracy for syndesmosis injuries without the need for CT imaging. The latter demonstrated lower sensitivity and specificity in this study. The distribution of fracture types was consistent with the previous literature, with lateral malleolus fractures being the most common isolated injury [14]. Supination-external rotation (SER) was identified as the most frequent injury pattern, aligning with the research of Lauge-Hansen et al. [26]. However, 10.6% of cases were unclassifiable using the Lauge-Hansen system, which is approximately double the rate reported in other studies [14].

The ankle mortise demonstrates an excellent biomechanical structure. The deltoid ligament and lateral malleolus maintain the optimal positioning of the talus relative to the tibia. Even a 1 mm lateral displacement of the talus can lead to a 42% change in joint contact, ultimately resulting in degenerative osteoarthritis [27]. Widening up to 5 mm in the medial clear space is considered normal in gravity-assisted stress ankle AP images for healthy ankles [23]. However, increased widening beyond 5 mm indicates a deep deltoid ligament injury with or without a concurrent syndesmosis injury. The gravitational stress applied to the ankle causes the weight of the talus to pull on the deltoid ligament and push on the fibula, leading to different scenarios depending on the type of fracture. For SER type II injuries, widening in the distal tibiofibular joint or at the medial clear space may not be expected. This is because the intact medial malleolus and/or deltoid ligament will prevent the talus from falling laterally. Bofelli et al. recommend obtaining at least three X-ray images under gravitational stress (AP, lateral, and mortise radiographs) to discriminate SER type II injuries from SER type IV injuries [12]. This issue is important because, despite the fact that these fractures are unstable, intervention for the syndesmosis is not always necessary, and overly aggressive treatment or unnecessary stabilization of the syndesmosis may lead to rigidity and pain during weight-bearing in the postoperative period [3].

In our series, 19 of 36 SER type II and IV cases did not involve syndesmotic injury (Figure 1), which is consistent with the idea that medial and lateral injuries must be carefully assessed before surgical stabilization. These findings highlight that GAASA imaging is particularly reliable in PER injuries, where all medial and lateral structures are disrupted and the fibula is extensively fractured. In such cases, gravitational force causes the talus to exert downward pressure on the distal fibular fragment, producing clear widening of the medial clear space and distal tibiofibular joint [28]. Maisonneuve injuries, not classified by the Lauge-Hansen system, are inherently unstable and require surgical syndesmosis stabilization [29]. In our study, three cases had clear distal tibiofibular widening without pathological medial clear space widening (Figure 2). This contrasts with the cadaveric study by Krahnebühl et al., which reported that stress radiographs are reliable only when deltoid and syndesmotic injuries coexist, but are ineffective for isolated syndesmosis injuries [5]. Our findings suggest that even when the medial malleolus and deltoid ligament remain intact, gravitational force alone can cause lateral fibular displacement, resulting in decreased tibiofibular overlap.

In unstable ankle injuries, GAASA imaging can reveal marked displacement, offering important diagnostic insights. However, these findings must be interpreted with caution. Even when distal widening is visible, proximal fibular alignment may indicate that the syndesmosis remains intact, and unnecessary stabilization could lead to postoperative stiffness or pain. Recent evidence reinforces this need for careful judgment. A prospective study on Weber B fractures showed that patients classified as “unstable” by gravity-stress radiographs but “stable” on weight-bearing films achieved comparable outcomes with nonoperative care, suggesting that gravity-stress imaging may overestimate instability when not correlated with functional loading [30]. Similarly, another comparative study demonstrated that weight-bearing radiographs provide a reliable basis for assessing stability and guiding nonoperative management in isolated lateral malleolar fractures, yielding excellent short-term clinical and radiographic outcomes. By contrast, gravity-stress radiographs tend to overestimate the need for surgical intervention [31]. Moreover, a 2024 review emphasized the lack of standardized protocols and the need for prospective multicenter validation before stress radiographs can be recommended for routine decision-making [32]. Compared to previous papers, our results reveal that GAASA imaging is a valuable and practical adjunct; however, the results must be interpreted in conjunction with a clinical and radiographic context to optimize diagnostic accuracy and guide appropriate surgical decision-making.

While axial CT images have provided reliable and specific assessments for syndesmosis injuries in previous studies, the diagnostic performance of CT was inferior to GAASA and mortise radiographs in this study [4]. Both inter-observer and intra-observer reliability revealed lower results compared to X-rays. This was an expected result, as the reliability of CT images is acceptable when volume measurement on axial images is performed at 10 mm over the plafond. In our study, the reason for evaluating the CT images subjectively without measurements was to mimic clinical practice scenarios where quantitative measurements are not always feasible. Relying solely on subjective evaluations of widening on axial CT images over the 10 mm plafond is potentially misleading. Even meticulous measurements on CT images do not guarantee accurate efficacy in detecting syndesmosis injuries. Yeung et al. [4] reported excellent inter-observer reliability for CT images in evaluating syndesmosis injuries. However, they found only a 56.4% sensitivity and 91.7% specificity on ROC curves, indicating the limitations of this approach. Despite excellent inter-observer reliability, the syndesmosis itself may not appear widened if unstressed or if any structures block the fibula from reducing to its proper position in CT imaging. When in a reduced position, the tibiofibular distance could appear within normal ranges on CT images and mortise radiographs (Figure 3) [1]. Researchers have proposed using direct arthroscopic visualization of the syndesmotic joint as a more precise diagnostic approach, as no imaging modality is perfect at detecting syndesmosis injuries [33]. From this perspective, some authors have utilized both conventional and dynamic ankle CT scans performed under forced external rotation, dorsiflexion, and axial loading in various foot positions. Their results revealed that applying stress significantly improves the diagnostic performance for detecting subtle syndesmotic instability [20,21]. To reduce radiation exposure and minimize the pain caused by stress applied to the ankle, ultrasonography (USG) has been utilized for the detection of ankle ligament injuries. It has been shown that ultrasound has good to excellent diagnostic value for complete discontinuity of the anterior talofibular ligament (ATFL) and the anterior inferior tibiofibular ligament (AITFL). Compared with static ultrasound, dynamic ultrasound demonstrated inferior diagnostic value for detecting complete discontinuity of the AITFL [34]. Although USG has high sensitivity and specificity in identifying ligamentous injuries such as the ATFL and AITFL, its role in assessing syndesmosis stability remains unclear. This is because the syndesmosis complex comprises five anatomical components, and in most cases, instability occurs only when all five structures are disrupted [31,35]. Furthermore, from an orthopedic surgeon’s perspective, the use of USG may not always be feasible or practical due to its operator-dependent nature, limited availability in some clinical settings, and the requirement for significant expertise to ensure accurate and reliable results.

This study has several limitations. First, its retrospective nature creates the potential for bias. The study population was limited to patients with suspected syndesmosis injury, which may have introduced selection bias and resulted in an overestimation of the diagnostic performance of the imaging modalities. To prevent this bias, a definitive diagnosis was established through a comprehensive evaluation process. For patients who underwent surgery, intraoperative notes regarding syndesmosis stability were reviewed; for those managed conservatively, follow-up X-rays were examined for signs of residual distal tibiofibular joint or widening of the medial clear space. In addition, we considered other clinical findings indicative of ankle instability alongside all available radiographic modalities to minimize diagnostic bias. In our series, two patients initially treated conservatively with a cast required subsequent surgical intervention after instability was detected during follow-up one week later. These patients were overlooked during the initial evaluation despite undergoing standard radiographs and CT imaging, as they presented with isolated lateral malleolus fractures at the Weber B level. However, none of the patients demonstrated clinical findings suggestive of ankle instability in either the surgically treated or conservatively managed groups, supporting the accuracy of our evaluation process for detecting syndesmosis instability. Additionally, this study did not assess the impact of these imaging techniques on clinical decision-making and patient outcomes, which would be an important next step to evaluate their true clinical utility.

## 5. Conclusions

In conclusion, gravity-assisted ankle stress AP (GAASA) radiography demonstrated encouraging diagnostic performance in this retrospective series, particularly with respect to sensitivity and negative predictive value. While GAASA may serve as a useful adjunct in evaluating syndesmosis injuries, its interpretation requires careful clinical correlation, and it should not be considered a replacement for standard imaging in all cases. GAASA may be particularly valuable in emergency or resource-limited settings where CT is not readily available, offering a practical option for ruling out injury in many patients. Nevertheless, when a syndesmosis injury cannot be confidently excluded, the combined use of mortise radiographs and CT scans remains the most reliable approach. Further prospective and multicenter investigations are warranted before systematic clinical implementation of GAASA can be recommended.

## Figures and Tables

**Table 1:** Patient demographics.

**Gender (*n*/%)**	Female	45	(37.2)
	Male	76	(62.8)
**Side (*n*/%)**	Right	59	(48.8)
	Left	62	(51.2)
**Anatomical classification (*n*/%)**	Isolated lateral malleolus fracture	57	(47.1)
	Isolated medial malleolus fracture	8	(6.6)
	Isolated posterior malleolus fracture	5	(4.1)
	Bimalleolar fracture	13	(10.7)
	Lateral + Posterior malleolus fracture	15	(12.4)
	Medial + posterior malleolus fracture	2	(1.7)
	Trimalleolar fracture	17	(14.0)
	Maisonneuve fracture	3	(2.5)
	Isolated ligamentous syndesmosis injury	1	(0.8)
**Weber class for lateral malleolus fracture (*n*/%)**	Weber A	15	(14.3)
	Weber B	67	(63.8)
	Weber C	23	(21.9)
**Treatment (*n*/%)**	Conservative	47	(38.8)
	Surgery	74	(61.2)
**Lauge-Hansen class (*n*/%)**	Supination external rotation 2	45	(42.1)
	Supination external rotation 3	13	(12.1)
	Supination external rotation 4	23	(21.5)
	Supination adduction 1	12	(11.2)
	Supination adduction 2	7	(6.5)
	Pronation external rotation 3	1	(0.9)
	Pronation external rotation 4	6	(5.6)

**Table 2:** Inter-observer and intra-observer reliability for the image sets.

**Fleiss’ Kappa for Inter-Observer Reliability**			
**Standard radiographs and mortise radiographs**			0.735
**Standard radiographs and GAASA images**			0.701
**Standard radiographs and CT axial and coronal images**			0.426
**Cohen’s kappa for intra-observer reliability**			
	**1st observer**	**2nd observer**	**3th observer**
**Standard radiographs and mortise radiographs**	0.911	0.706	0.461
**Standard radiographs and GAASA images**	0.833	0.733	0.563
**Standard radiographs and CT axial and coronal images**	0.15	0.06	0.19

GAASA image: gravity-assisted ankle stress AP (GAASA) image. AP: anterior–posterior radiographs; 1st observer, senior resident surgeon; 2nd observer, staff orthopedic surgeon; 3rd observer, senior orthopedic surgeon.

**Table 3:** The need for further CT images for different surgeons at various seniority levels.

	Senior Resident Surgeon	Staff Orthopedic Surgeon	Senior Orthopedic Surgeon
**Standard radiographs and mortise radiographs**	56 (46.3%)	26 (21.3%)	12 (9.9%)
**Standard radiographs and GAASA images**	37 (30.6%)	6 (5%)	7 (5.8%)

GAASA image: gravity-assisted ankle stress AP (GAASA) image. AP: anterior–posterior radiographs.

## Data Availability

The datasets used and/or analyzed during the current study are available from the corresponding author on reasonable request.

## Supplementary Materials

The following supporting information can be downloaded at: https://www.mdpi.com/article/10.3390/diagnostics15212803/s1, Table S1: Parameters showing effectiveness of different image sets.

## Abbreviations

The following abbreviations are used in this manuscript:

GAASA	Gravity-Assisted Ankle Stress AP
CT	Computed Tomography
AP	Anterior–Posterior
Lat	Lateral
USG	Ultrasonography
AITFL	Anterior Inferior Tibiofibular Ligament
ATFL	Anterior Talofibular Ligament
