# Supplementary material for: Evaluating the Effectiveness of Gravity-Assisted Ankle Stress AP Imaging in Detecting Syndesmosis Injuries: A Retrospective Clinical Study
Source: Diagnostics (Basel). 2025 Nov 5;15(21):2803. doi: 10.3390/diagnostics15212803 (PMC12610165; doi:10.3390/diagnostics15212803)
Supplement: Supplementary file 1 [file diagnostics-15-02803-s001.zip › diagnostics-3835971-supplementary.pdf]

Supplementary Table S1: Parameters showing effectiveness of different image sets

		Sensitivity%	Specificity%	Positive predictive value%	Negative predictive value%
Efficacy of Different Imaging Sets Based on Evaluator Experience					
**Senior resident**	**Standard radiographs and Mortise radiographs**	77,3	81,4	68	87
	**Standard radiographs and GAASA images**	71,4	71,4	55,6	83,3
	**Standard radiographs and CT axial and coronal images**	36,8	63,7	32,6	68
**Staff orthopaedic surgeon**	**Standard radiographs and Mortise radiographs**	63,6	66,1	50	77,4
	**Standard radiographs and GAASA images**	74,4	53,9	45	80,4
	**Standard radiographs and CT axial and coronal images**	39,5	52,5	28,3	64,6
**Senior orthopaedic surgeon**	**Standard radiographs and Mortise radiographs**	50	84	58	78
	**Standard radiographs and GAASA images**	77,1	67,1	50,9	86,9
	**Standard radiographs and CT axial and coronal images**	36,8	43,8	23,7	59,3
Efficacy of Different Imaging Sets Based on Overall diagnosis					
		**Sensitivity%**	**Specificity%**	**Positive predictive value%**	**Negative predictive value%**
	**Standard AP/lateral radiographs and Mortise radiographs**	55,3	81,5	58,3	79,5
	**Standard AP/lateral radiographs and GAASA images**	82,1	65,9	53,3	88,5
	**Standard radiographs and CT axial and coronal images**	34,2	53,8	26	63

Overall diagnosis: the option selected at the first session by a two-thirds majority in the radiograph sets for each patient
